# Retraction: Experimental orthotopic transplantation of a tissue-engineered oesophagus in rats

**DOI:** 10.1038/ncomms15077

**Published:** 2017-03-21

**Authors:** Sebastian Sjöqvist, Philipp Jungebluth, Mei Ling Lim, Johannes C. Haag, Ylva Gustafsson, Greg Lemon, Silvia Baiguera, Miguel Angel Burguillos, Costantino Del Gaudio, Antonio Beltrán Rodríguez, Alexander Sotnichenko, Karolina Kublickiene, Henrik Ullman, Heike Kielstein, Peter Damberg, Alessandra Bianco, Rainer Heuchel, Ying Zhao, Domenico Ribatti, Cristián Ibarra, Bertrand Joseph, Doris A. Taylor, Paolo Macchiarini

Nature Communications 5: Article number: 3562; DOI: 10.1038/ncomms4562 (2014); Published 04 15 2014; Updated 03 21 2017

This Article is retracted by the authors. *Nature Communications* previously issued an Editorial Expression of Concern (http://www.nature.com/articles/ncomms13310) related to this Article, following the publication of a report commissioned by The Karolinska Institute and prepared by the Expert Group for Misconduct in Research at the Swedish Central Ethical Review Board. Owing to the technical issues raised by this investigation, we, the authors, are now retracting the Article.

P.M., S.S., Y.G., S.B., M.A.B., C.D.G., A.S., K.K., H.K., P.D., A.B., R.H., D.R., C.I., B.J. and D.A.T. agree with this retraction. P.J., M.L.L., J.H., G.L., A.B.R., H.U. and Y.Z. could not be reached for comment.

